# Fat Embolism Syndrome Following Elective Orthopedic Surgery in a Patient With Duchenne Muscular Dystrophy

**DOI:** 10.7759/cureus.112419

**Published:** 2026-07-10

**Authors:** Kareem Hassan, Ravi Desai, Emir Shahin, Peter Shapiro, Ofer Burshtain, Daniel Dragone

**Affiliations:** 1 Anesthesiology, Hackensack University Medical Center, Hackensack, USA; 2 Anesthesiology, Rowan-Virtua School of Osteopathic Medicine, Stratford, USA

**Keywords:** achilles tendon lengthening, acute ischemic stroke anesthesia, becker muscular dystrophy, duchenne muscular dystrophy (dmd), fat embolism syndrome (fes), hypoxic-ischemic encephalopathy (hie)

## Abstract

Duchenne muscular dystrophy (DMD) is associated with substantial perioperative morbidity due to progressive cardiopulmonary disease and altered muscle physiology; however, fat embolism syndrome (FES) is rarely considered during perioperative risk assessment for this population. We report the case of an 11-year-old boy with genetically confirmed DMD, chronic corticosteroid therapy, severe osteopenia, and a patent foramen ovale who developed fulminant multisystem FES following elective bilateral Achilles tendon lengthening. Despite guideline-concordant anesthetic management using total IV anesthesia with avoidance of triggering agents, the patient developed acute hypoxemic respiratory failure and progressive encephalopathy within hours of surgery, followed by seizures, retinal microemboli, and diffuse pulmonary infiltrates. Extensive evaluation excluded infectious, inflammatory, and primary vascular etiologies, and the patient met multiple major and minor Gurd’s criteria for FES. This case highlights that children with DMD may be uniquely susceptible to FES even after seemingly low-risk elective orthopedic procedures, independent of anesthetic technique or intraoperative instability. It underscores the need for heightened postoperative vigilance and disease-specific perioperative risk stratification in this vulnerable population.

## Introduction

Duchenne muscular dystrophy (DMD) is a severe X-linked recessive neuromuscular disorder caused by mutations in the dystrophin gene, resulting in the absence of dystrophin, a protein essential for maintaining sarcolemmal integrity during muscle contraction. Loss of dystrophin leads to repeated myofiber injury, calcium influx, and progressive muscle degeneration with replacement by fat and connective tissue. Clinically, DMD presents in early childhood with progressive proximal weakness, calf pseudohypertrophy, and markedly elevated creatine kinase levels, followed by loss of ambulation and eventual cardiopulmonary failure, which remains the leading cause of mortality [1].

Glucocorticoids are a cornerstone of DMD management, shown to prolong ambulation, delay scoliosis, and preserve respiratory and cardiac function [2,3]. However, chronic steroid exposure during childhood has profound skeletal consequences. Glucocorticoids impair osteoblast activity, suppress bone formation, and disrupt normal bone accrual, resulting in reduced bone mineral density and osteoporosis. DMD itself already confers a baseline risk for fractures due to progressive muscle weakness, reduced mechanical stimulation of bone, and systemic inflammation, with studies showing a fourfold higher fracture incidence than in healthy individuals [4]. This risk increases further with long-term glucocorticoid therapy, with a 16-fold higher risk of first fracture than in steroid-naive boys with DMD [5].

Long-bone fractures are a well-established trigger for fat embolism syndrome (FES), a rare but potentially fatal condition characterized by acute hypoxemic respiratory failure, neurologic dysfunction, and systemic manifestations resulting from the dissemination of fat globules into the pulmonary and systemic microvasculature [6]. Patients with DMD may be uniquely susceptible to FES due to the convergence of severe osteopenia, chronic steroid use, expanded fatty marrow, and increased fracture risk. Intracardiac shunts, such as a patent foramen ovale (PFO), may further facilitate paradoxical cerebral embolization, leading to disproportionate neurologic injury.

Despite these vulnerabilities, FES is rarely considered in the perioperative risk assessment of patients with DMD, particularly following elective or ostensibly low-risk orthopedic procedures. We report the case of an 11-year-old boy with DMD who developed severe multisystem FES following elective Achilles tendon lengthening. This report describes the clinical course, diagnostic evaluation, and perioperative implications of fulminant FES after elective Achilles tendon lengthening in a child with DMD.

## Case presentation

An 11-year-old boy with genetically confirmed DMD was admitted for elective bilateral gastrocnemius-soleus complex tendon lengthening to improve comfort, positioning, and tolerance of ankle-foot orthoses. He had been wheelchair-dependent since age 7 and was receiving comprehensive neuromuscular care, including daily prednisone (0.75 mg/kg for five years), prior exon-skipping therapy with eteplirsen (Exondys 51), and more recent treatment with Elevidys micro-dystrophin gene therapy. His medical history was notable for steroid-associated osteopenia (DEXA Z-score, -2.5), mild obstructive sleep apnea treated with nocturnal noninvasive ventilation, and a small PFO identified on bubble echocardiography. He had no history of heart failure, hypoventilation, or prior anesthetic complications. Preoperative echocardiography and pulmonary function testing were within expected limits for DMD.

The patient underwent bilateral open Achilles tendon Z-lengthening with short-leg cast application under general anesthesia. Anesthetic management followed DMD-specific guidelines, including induction and maintenance with total IV anesthesia using propofol and remifentanil, without volatile agents or depolarizing neuromuscular blockers. Endotracheal intubation was atraumatic, neuromuscular blockade was limited to small doses of rocuronium, and bilateral popliteal nerve blocks were placed for postoperative analgesia. Intraoperative vital signs remained stable throughout, with oxygen saturation >98% on FiO₂ 0.5 and no episodes of hypotension, arrhythmia, or hypercarbia.

Following surgery, he was admitted to the PICU, where he arrived markedly somnolent and required a non-rebreather mask for oxygenation. Although initially stable, he developed worsening hypoxemia within hours, with oxygen saturation declining into the 80% range while still sedated. This prompted escalation to bilevel positive airway pressure, along with albuterol and chest physiotherapy to support airway clearance and optimize lung function. Opioid analgesics were avoided, and pain control was maintained with IV acetaminophen and ketorolac. Despite stable arterial blood gas results and the avoidance of opioid analgesics, the patient failed to regain consciousness, opening his eyes only briefly in response to sternal rub.

**Figure 1:**
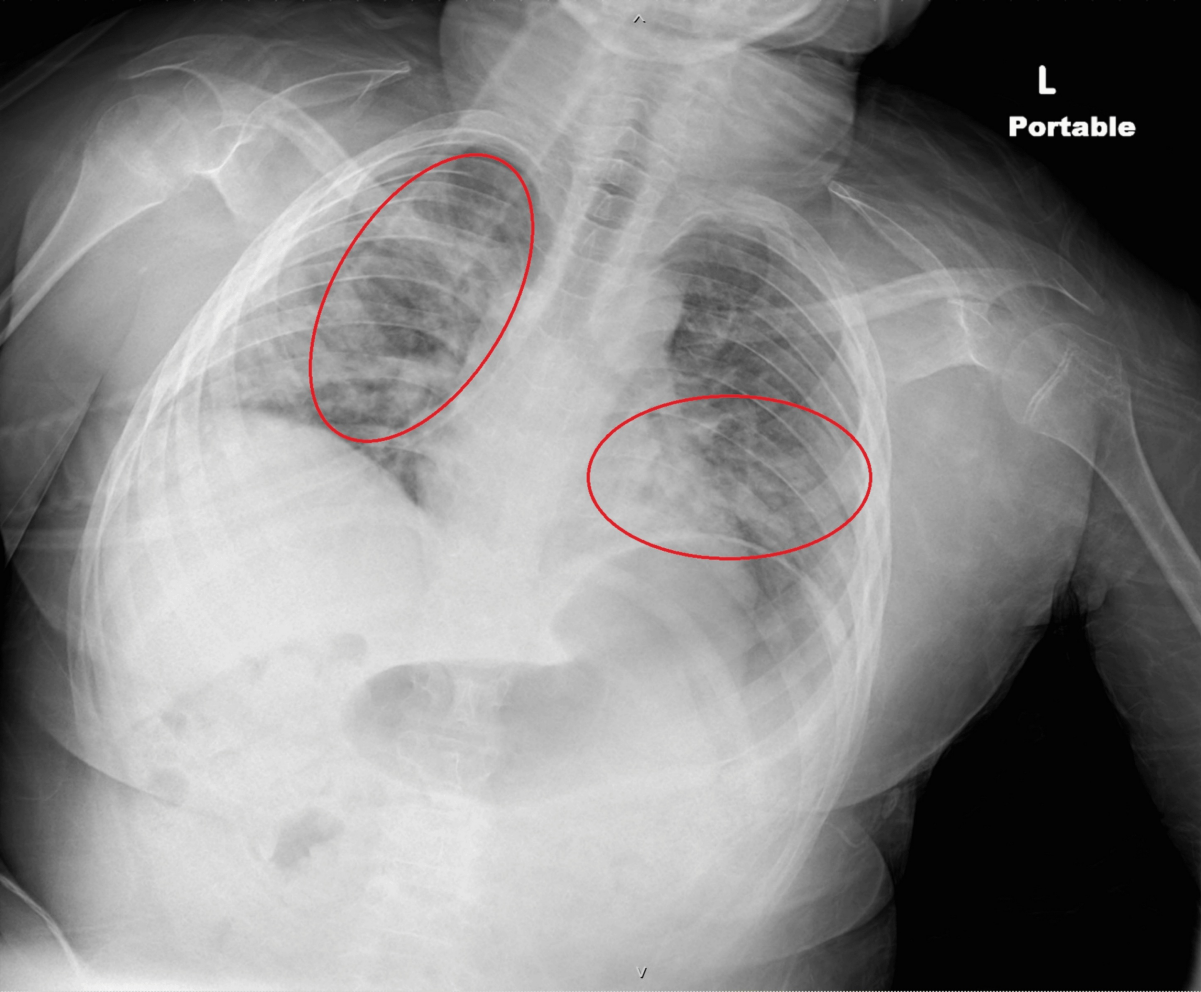
Chest X-ray showing bilateral low lung volumes and patchy pulmonary parenchymal opacities (red circles)

By postoperative day (POD) 1, the patient remained obtunded, responding only to noxious stimuli. Chest radiography revealed diffuse bilateral pulmonary infiltrates (Figure 1). He subsequently experienced a generalized tonic-clonic seizure, which was treated with IV lorazepam. Continuous electroencephalography demonstrated diffuse background slowing without ongoing epileptiform activity. A non-contrast head CT was unremarkable. Given concern for metabolic, infectious, or inflammatory etiologies, a comprehensive diagnostic evaluation was initiated.

**Figure 2:**
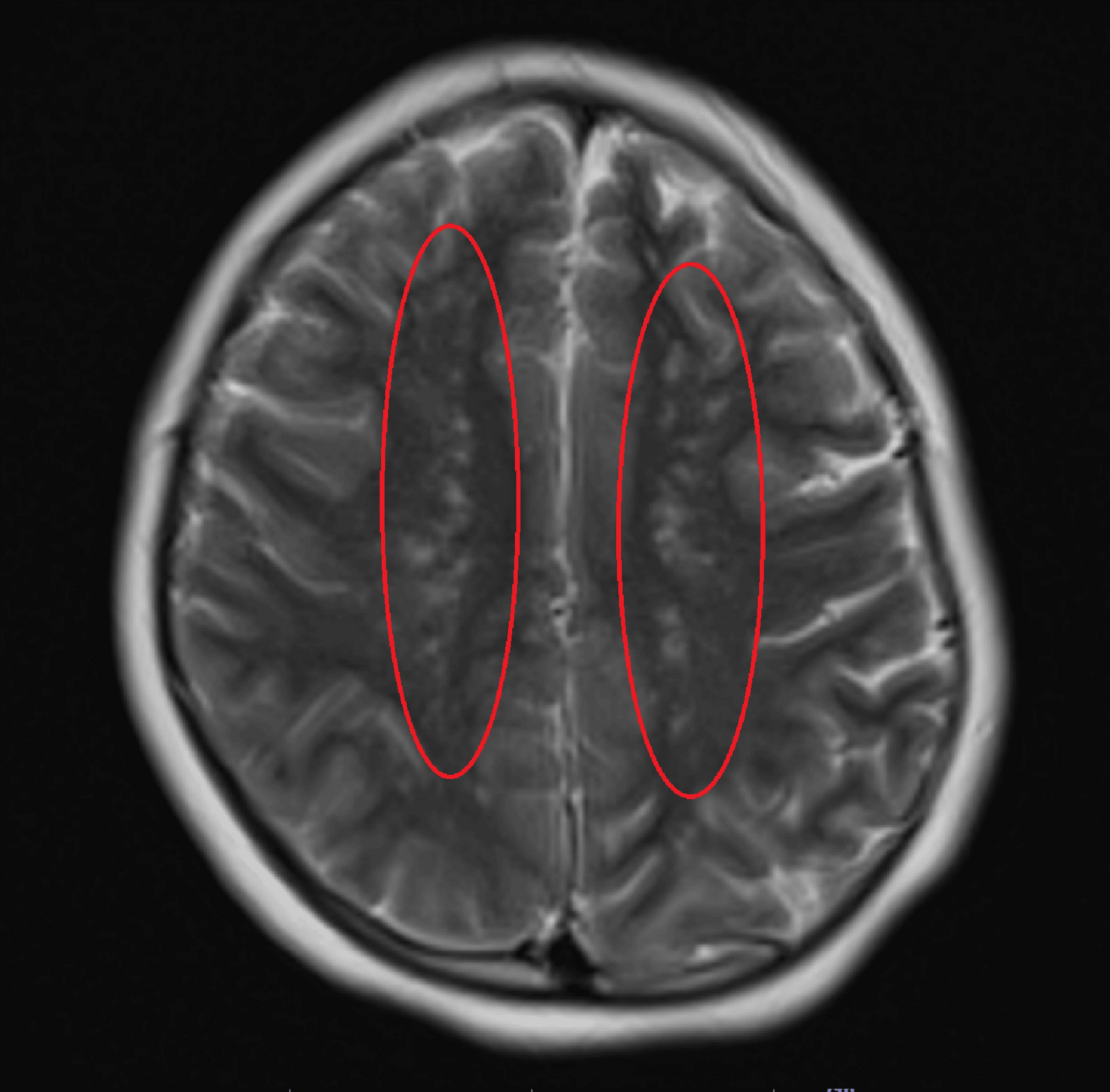
Axial diffusion-weighted MRI showing innumerable foci of bright diffusion (red circles), demonstrating bilateral supratentorial watershed infarcts in the ACA-MCA border zones consistent with hypoperfusion injury MRI: magnetic resonance imaging, ACA-MCA: anterior cerebral artery-middle cerebral artery

**Figure 3:**
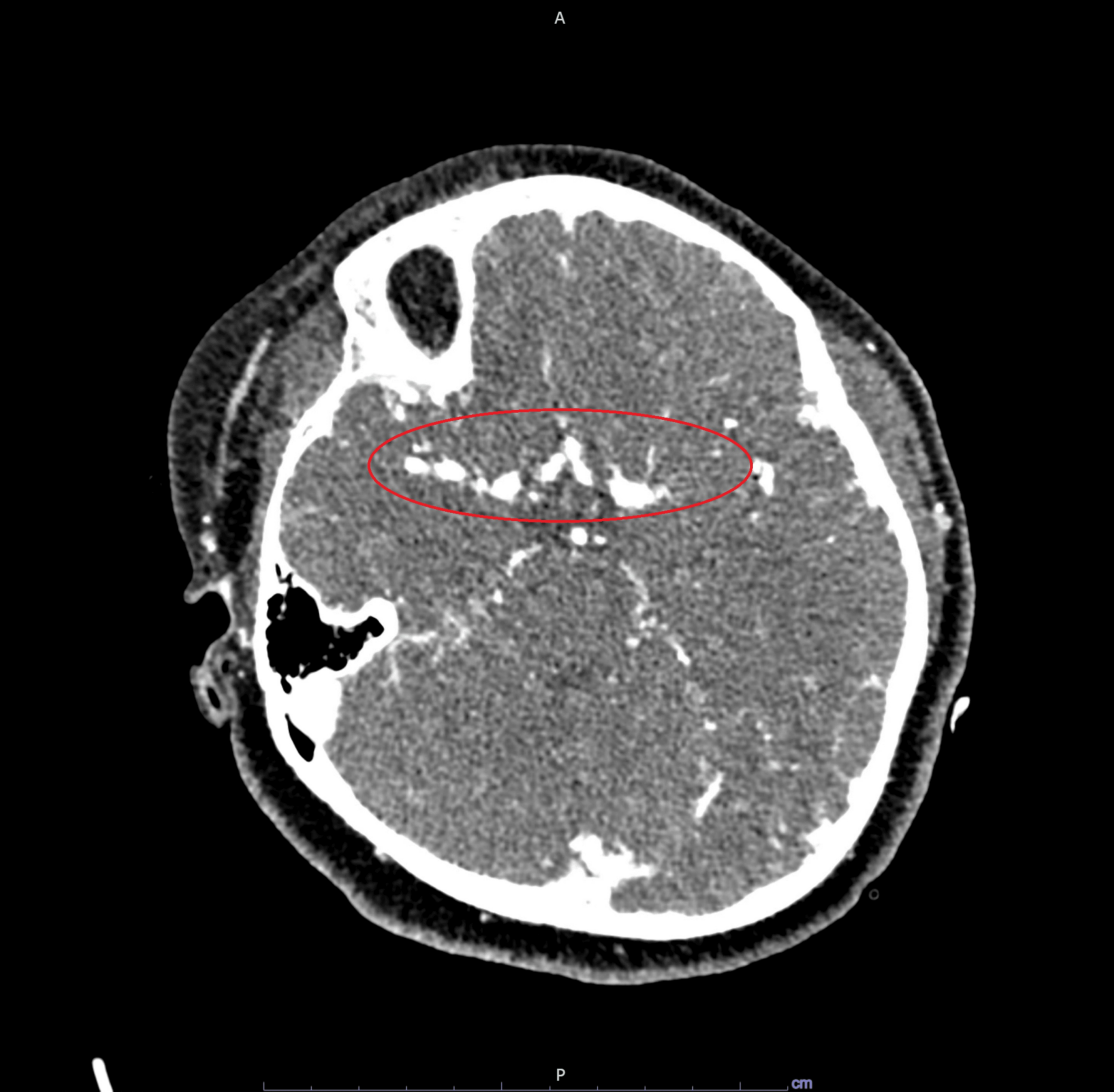
Contrast-enhanced CT angiogram showing irregular appearances of the left and right proximal MCA branches (red circle), initially concerning for vasculitis versus RCVS CT: computed tomography, MCA: middle cerebral artery, RCVS: reversible cerebral vasoconstriction syndrome

On POD 2, brain MRI with diffusion-weighted imaging demonstrated multiple scattered punctate areas of diffusion restriction in the bilateral anterior cerebral artery/middle cerebral artery watershed regions, with additional cortical and cerebellar involvement (Figure 2). MR angiography and subsequent CT angiography revealed segmental vessel irregularities described as a "string-of-beads" appearance, raising initial concern for vasculitis or reversible cerebral vasoconstriction syndrome (RCVS) (Figure 3). A repeat bubble echocardiogram confirmed right-to-left shunting through the known PFO. Extensive evaluation, including cerebrospinal fluid analysis, infectious PCR testing, inflammatory markers, autoimmune serologies, thrombophilia testing, and transthoracic echocardiography, failed to identify an alternative etiology.

**Figure 4:**
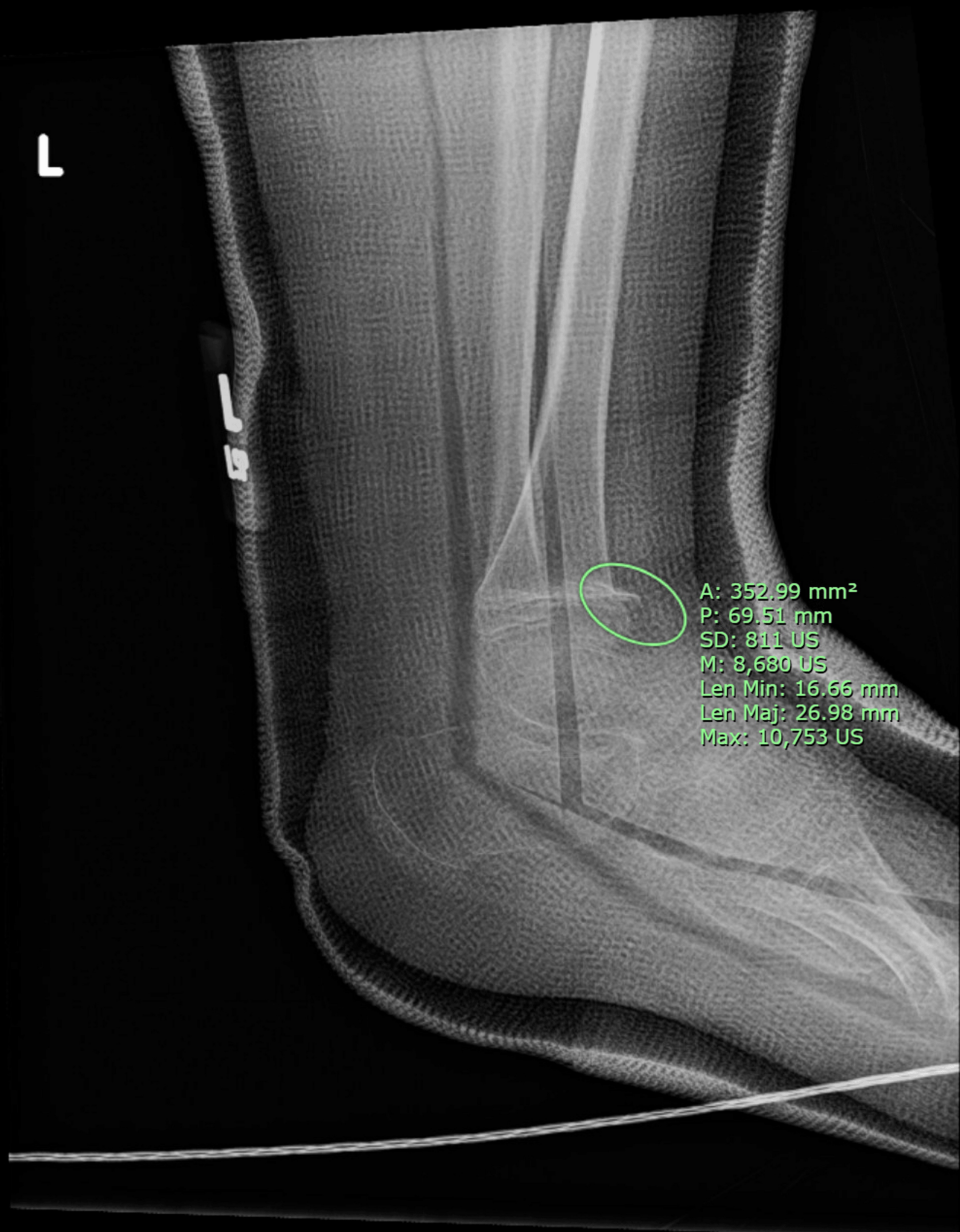
Left ankle X-ray showing a nondisplaced buckle fracture of the anterior metaphysis of the distal tibia

By POD 4, diagnostic cerebral angiography demonstrated no evidence of vasculitis or RCVS. Reevaluation of the neuroimaging findings favored a hypoxic-ischemic or microembolic process. A nondisplaced distal tibial fracture was identified on postoperative radiographs, likely sustained perioperatively in the setting of osteopenia (Figure 4). On POD 5, ophthalmologic examination revealed retinal petechiae consistent with microembolic injury. Concurrent chest imaging showed mild patchy ground-glass opacities.

Given the constellation of acute hypoxemia, encephalopathy with seizures, retinal microemboli, pulmonary infiltrates, anemia, thrombocytopenia, and fever occurring after recent orthopedic surgery in the setting of an occult long-bone fracture, chronic steroid use, osteopenia, and a PFO, a diagnosis of FES was made.

Supportive management was continued, including mechanical ventilation, osmotic therapy for cerebral edema, and antiseizure treatment with levetiracetam. High-dose corticosteroids were avoided because of concern that they could exacerbate marrow fat release. Over the subsequent days, the patient demonstrated gradual neurologic improvement. By POD 6-7, he was awake, following simple commands, and was successfully extubated to noninvasive ventilation. At one-month follow-up, he exhibited mild residual spasticity and cognitive slowing but continued to recover.

## Discussion

This case illustrates a rare but clinically significant presentation of FES following elective orthopedic surgery in a child with DMD. Although FES is classically associated with high-energy long-bone fractures and polytrauma, this report demonstrates that patients with DMD may develop FES after seemingly low-risk orthopedic procedures that do not involve long bones. The case challenges conventional assumptions regarding perioperative safety in DMD. It highlights how disease-specific physiology fundamentally alters risk profiles, rendering traditional trauma-based frameworks for FES incomplete in this population.

Long-term corticosteroid therapy in DMD, while disease-modifying, disrupts normal bone formation and accelerates osteoporosis during critical periods of skeletal development, resulting in a markedly increased fracture risk even after minimal mechanical stress [4,5]. This vulnerability is most pronounced in non-ambulatory patients, who exhibit severe osteopenia and fatty marrow infiltration, creating conditions under which minor orthopedic manipulation or perioperative stress may be sufficient to mobilize marrow fat [7]. Prior reports support this lowered threshold, including Stein et al., who described FES following minor trauma in a child with DMD, and Murphy et al., who emphasized steroid-associated skeletal fragility as a key predisposing factor in two survivors of DMD-associated FES [8,9]. Importantly, Specht et al. reported a case of FES in a patient with DMD who underwent elective orthopedic surgery and had never received corticosteroid treatment, underscoring that the pathophysiology of DMD itself may be sufficient to predispose patients to FES [10]. In our case, the clinical and radiographic findings most strongly support embolization of marrow fat from steroid-compromised bone, with dystrophic, fat-infiltrated muscle likely contributing additional embolic material during surgical manipulation rather than serving as the primary embolic source. Taken together, these observations support reframing FES in DMD as a disease- and treatment-mediated complication rather than a purely trauma-mediated event.

Importantly, the clinical consequences of FES in DMD may be substantially more severe than those in otherwise healthy children, with reported mortality approaching 30% and nearly half of survivors failing to achieve full neurologic recovery, compared with approximately 5% mortality and more than 90% full recovery in the general pediatric trauma population with long-bone fractures [10]. These outcome disparities further support the concept that DMD confers not only a lower threshold for embolization but also diminished physiologic reserve to tolerate embolic insults.

The presence of a PFO in this patient provides a plausible mechanism for paradoxical embolization contributing to the observed neurologic sequelae. Fat emboli lodge within the pulmonary microvasculature, causing mechanical obstruction and triggering a severe inflammatory response. This leads to increased pulmonary vascular resistance and acute right-heart strain, which can unmask a previously hemodynamically insignificant PFO, creating a functional right-to-left shunt and allowing systemic dissemination of embolic material to the cerebral and retinal circulations [11]. However, a PFO is not required for the development of cerebral fat embolism, as fat globules may traverse the pulmonary capillary bed directly under conditions of high embolic load or pulmonary vascular stress [12]. The patient's multifocal bilateral watershed infarcts, retinal microembolic findings, encephalopathy, and seizures in the absence of large-vessel occlusion are consistent with cerebral fat embolism. Accordingly, although the PFO may have amplified the neurologic injury in this case, it should be regarded as a risk modifier rather than a prerequisite, reinforcing that patients with DMD remain vulnerable to systemic fat embolization even in the absence of intracardiac shunting.

An additional challenge highlighted by this case is the diagnostic uncertainty that can characterize early postoperative neurologic deterioration. Initial neuroimaging raised concern for primary central nervous system vasculitis or RCVS, diagnoses that have been reported following physiologic stress in the postoperative setting. CT angiography demonstrated segmental vessel irregularities, and MRI revealed diffusion-restricted lesions that overlapped with patterns seen in hypoxic-ischemic injury. However, subsequent diagnostic cerebral angiography was normal, inflammatory and autoimmune studies were unrevealing, and multidisciplinary review favored hypoxic-ischemic or embolic injury rather than inflammatory vasculopathy. This diagnostic trajectory highlights an important pitfall: fat embolism can mimic vasculitis, RCVS, or hypoxic-ischemic encephalopathy, particularly when neurologic manifestations predominate. Awareness of this mimicry is critical to avoid unnecessary immunosuppression or invasive testing and to focus on supportive management.

An important limitation is that the distal tibial fracture was radiographically occult in the immediate postoperative period, and its exact timing cannot be definitively established. However, the absence of preoperative pain, deformity, or neurologic symptoms, coupled with the abrupt postoperative onset of hypoxemia and encephalopathy following orthopedic manipulation and casting, makes a perioperative fracture the most plausible explanation in the setting of severe steroid-induced osteopenia. Moreover, FES has been described in patients without high-energy trauma or radiographically evident fracture, supporting the possibility that minimal mechanical stress alone may trigger marrow fat mobilization [13]. Accordingly, delayed fracture recognition does not weaken the diagnosis; rather, it underscores the uniquely low threshold for embolization in this population.

FES lacks specific laboratory markers and therefore relies on clinical criteria supported by imaging findings, necessitating a high index of suspicion in at-risk patients. In this setting, Gurd's clinical criteria (Table 1) remain a useful diagnostic framework when applied judiciously in atypical presentations [14]. In the present case, the patient met multiple major criteria, including acute hypoxemia, central nervous system depression, and retinal changes, as well as several minor criteria, including anemia, thrombocytopenia, renal dysfunction, and pulmonary infiltrates, supporting the diagnosis of FES despite the absence of the typical precipitating event of overt long-bone trauma [15]. Notably, non-trauma populations remain underrepresented in the FES literature, and this case extends the applicability of established diagnostic criteria to children with neuromuscular disease undergoing elective surgery.

**Table 1:** Gurd's criteria for diagnosing FES Diagnostic criteria: The diagnosis is established by the presence of at least one major criterion and four minor criteria, or two major criteria. PaO₂: partial pressure of arterial oxygen, AMS: altered mental status, ESR: erythrocyte sedimentation rate, FES: fat embolism syndrome

Major criteria	Minor criteria
1. Petechial rash	1. Tachycardia (>110 bpm)
2. Respiratory insufficiency (PaO₂ < 60 mmHg)	2. Fever (>38.5°C)
3. Cerebral involvement (confusion, AMS, seizures)	3. Retinal changes
-	4. Jaundice
-	5. Renal dysfunction
-	6. Anemia (acute drop in hemoglobin)
-	7. Thrombocytopenia
-	8. Elevated ESR
-	9. Fat macroglobulinemia

From an anesthetic perspective, this case is particularly instructive because it occurred despite strict adherence to DMD-specific anesthetic guidelines. Triggering agents such as succinylcholine and volatile anesthetics were avoided; total IV anesthesia was used; neuromuscular blockade was minimized; and intraoperative hemodynamics remained stable. These measures are essential for preventing rhabdomyolysis, hyperkalemia, and malignant hyperthermia-like reactions in DMD; however, they do not mitigate disease-intrinsic risks such as fat embolization. For anesthesiologists, this case underscores that even guideline-concordant anesthetic management cannot eliminate the profound systemic vulnerabilities imposed by DMD and its treatments. Postoperative neurologic or respiratory deterioration therefore should not be attributed reflexively to anesthetic drug effects alone.

This case also has important implications for perioperative planning, risk stratification, and informed consent in children with DMD. Elective orthopedic procedures in this population are frequently performed to improve comfort, positioning, or orthotic tolerance rather than functional recovery. However, the present case demonstrates that even procedures traditionally considered low risk may confer disproportionate systemic risk in the setting of advanced disease, chronic corticosteroid exposure, and skeletal fragility. Accordingly, preoperative evaluation should extend beyond standard cardiopulmonary assessment to include consideration of bone health, cumulative corticosteroid exposure, and factors that may amplify embolic risk, such as intracardiac shunting. Preoperative counseling should explicitly address the possibility of rare but severe complications, including FES, and perioperative planning may benefit from multidisciplinary input and heightened postoperative monitoring, even for procedures typically considered low risk. Finally, as a single-case report with retrospective clinical interpretation, the precise contribution of each proposed mechanism, including the occult fracture, surgical manipulation, and the PFO, to the development and severity of FES cannot be determined definitively.

## Conclusions

This case broadens the recognized spectrum of FES to include low-risk elective orthopedic surgery in children with DMD. It highlights how disease-specific physiology and treatment-related skeletal vulnerability, including chronic corticosteroid exposure and bone fragility, can lower the threshold for catastrophic fat embolic events, independent of anesthetic technique or intraoperative instability. Despite guideline-concordant anesthetic management, anesthesia-related safety measures alone cannot fully mitigate the intrinsic systemic risks associated with DMD. Clinicians should therefore maintain a high index of suspicion for FES when postoperative hypoxemia or unexplained neurologic deterioration occurs, even in the absence of an overt long-bone fracture or intraoperative instability. Comprehensive preoperative risk assessment, early recognition, and multidisciplinary perioperative management are essential to improving outcomes in this uniquely vulnerable population.
